# Precarious employment and migrant workers’ mental health: a protocol for a systematic review of observational studies

**DOI:** 10.1186/s13643-020-01313-w

**Published:** 2020-03-07

**Authors:** Ozlem Koseoglu Ornek, Tobias Weinmann, Julia Waibel, Katja Radon

**Affiliations:** 1 Occupational and Environmental Epidemiology & NetTeaching Unit, Institute and Clinic for Occupational, Social and Environmental Medicine, University Hospital, LMU Munich, Ziemssenstr. 1, 80336 Munich, Germany; 2 Department of Nursing, Faculty of Health Sciences, Istanbul Bilgi University, Dolapdere Kampus, Hacıahmet Mahallesi, Pir Hüsamettin Sokak No: 20, 34440 Beyoğlu, Istanbul, Turkey

**Keywords:** Migrants, Immigrant, Immigrant worker, Precarious, Temporary employment, Occupational health, Systematic review protocol

## Abstract

**Background:**

Precarious employment has become an urgent public health issue at a global scale with potential consequences for quality of life and health of employees, especially in vulnerable groups such as migrants. The primary aim of this systematic review is thus to analyze and summarize existing research on the association between precarious employment and migrant workers’ mental health.

**Methods:**

We will search PubMed/MEDLINE, PsycINFO, Web of Science (from January 1970 onwards) for original articles on observational studies (e.g., cohort, case-control and cross-sectional, and qualitative) published in English, German, Turkish, and Spanish. The primary outcome will be depression and anxiety disorders. Secondary outcomes will be burnout, sleeping problems, and occupational stress. Two reviewers will independently screen all citations, full-text articles, and abstract data. Potential conflicts will be resolved through discussion. The methodological quality (or risk of bias) of individual studies will be appraised using an appropriate tool. A narrative synthesis will summarize and explain the characteristics and findings of the studies. If feasible, we will conduct random effects meta-analyses where appropriate.

**Discussion:**

This systematic review will analyze the ways in which precarious employment affects migrant workers’ mental health and the process that underlies this relationship. The results from the systematic review outlined in this protocol will be of interest to labor and health professionals, policy makers, labor unions, and non-governmental organizations. Our findings may encourage and impel related policy makers to establish human-focused, safe and healthy work environments, and workplace conditions.

**Systematic review registration:**

PROSPERO, CRD42019132560

## Background

Across the world, precarious employment has become a common phenomenon during the last several decades [1]. Although its definition of may vary according to the country, economy, labor markets, and social policy, it is commonly related to one or more of the following terms: temporary, atypical, contingent, or non-standard work; job insecurity; lack of work rights; and inadequate salary. In addition, new forms of precarious employment are currently emerging as a consequence of the rise of the so-called “gig economy.” Gig work is a type of precarious employment in which consumers connect with workers offering services (“gigs”) such as cleaning, cooking, food delivery, and transportation (e.g., Uber) through online platforms [2, 3]. Workers in this economy often have nonstandard arrangements that translate to the following: low payment, unstable, part-time, or temporary work contacts, and no or little access to workplace rights and social benefits [2, 4]. It is common among vulnerable groups, such as young workers who experience a higher unemployment rate and workers who earn insufficient salaries [2]. Many factors play a role in the development of these kinds of precarious work conditions, but globalization, neoliberal politics, and regression of social policies are the main factors contributing to precarious employment [5–8].

In addition to becoming even more prevalent and visible in recent decades, precarious work has been linked by various studies to perceived poor health and quality of life among workers [9, 10]. Migrant workers who are generally regarded as a vulnerable group [11, 12] are considered to be especially affected by precarious employment conditions [9]. They are more likely to be exposed to hazards in the workplaces due to low awareness of working rights, language barriers, powerlessness to demand or implement workplace rights, and insufficient occupational health and service policies [12–14]. Additionally, migrants are more likely to face various challenges in the new setting to access quality employment. According to scientific studies, prejudices by employers [15], lack of professional networks [16], and language barriers [17] are the main relevant factors. Additionally, many studies showed that precarious employment conditions are linked to mental health problems such as depression, anxiety, burnout, occupational stress [18], occupational injuries, and accidents among migrant workers [19–21]. However, although a variety of reviews have been published recently on the association between precarious employment and health, including mental health, mostly in high-income countries [7, 8, 22–25], to our knowledge, there has been no systematic review on the association between precarious employment and migrant workers’ health. Thus, there is a need to better understand and systematically evaluate the effect of precarious work conditions on migrants’ mental health.

The main aim of our planned systematic review of qualitative and quantitative studies is to analyze and summarize existing research on the association between precarious employment and migrant workers’ mental health. The review of qualitative and quantitative studies specifically will focus on answering the following scientific research questions.

Quantitative studies:

- What is the frequency of precarious employment among migrants?
- What is the association between precarious employment and mental health, including its direction and effect size?
- Qualitative studies:
- What are the experiences of precarious employment among migrants?
- What processes underlie the relationship between precarious employment and migrant workers’ mental health?

## Methods/design

This study protocol is being reported in accordance with the reporting guidance the Preferred Reporting Items for Systematic Reviews and Meta-Analyses Protocols (PRISMA-P) statement [26] (see PRISMA-P checklist in Additional file 1). It was registered within the International Prospective Register of Systematic Reviews (PROSPERO) (registration number: CRD42019132560). Significant protocol amendments will be reported with the publication of the review.

### Eligibility criteria

The eligibility criteria of the included publications will be described based on the Population, Exposure, Comparator, and Outcome (PECO) framework [27].

- Types of population: We will include original studies of working-age (≥ 15 years) migrant workers in the formal and informal economy. Participants residing in any country and from any occupation or industry will be included. We will exclude studies in which participants are students, children below working age (< 15 years), and internal migrant workers.
- Types of exposure: We will include studies that defined “precarious employment” or work conditions that are defined as precarious as their exposure variable. We will include all original studies where “precarious work conditions” were measured (quantitative studies) or where the relationships between themes were supported by at least one quotation or author interpretation (qualitative studies).
- Types of **c**omparators: The main comparator will be no/lower exposure to the described components of precarious work conditions.
- Types of outcomes: The primary outcomes of interest are depression and anxiety disorders, the secondary outcomes are burnout, sleeping problems, and occupational stress. The results of the included studies will be classified for those outcomes.
- Study design: We will include original studies of qualitative and quantitative observational studies (cross-sectional, case-control, and cohort studies). Case studies, editorial letters, conference papers, dissertations, expert opinions, letters to the editor, commentaries, and reviews will be excluded.
- Setting: No restriction by the type of setting will be used.
- Time of publication: All articles published from January 1, 1970 onwards will be included.
- Publication status: Articles that are published in peer-reviewed journals and e-publications ahead of print will be included.
- Language: Study records written in English, German, Spanish, and Turkish will be included.

### Information sources

We will perform a search of the current literature using the electronic databases PubMed/Medline, PsycINFO, and Web of Science, with the inclusion of the Science Citation Index Expanded, Social Sciences Citation, and Art and Humanities Citation Index databases. We will search by hand for potential relevant studies in the following:

- Reference lists of all included studies,
- Reference lists of previously published related systematic reviews,
- Study records published in the last 12 months in the four peer-reviewed academic journals from which we obtain the largest number of included articles, and
- The PubMed/Medline database for the most recent publications, including e-publications ahead print over the last 2 months, just before completing the review.
- Websites of relevant organizations such as the World Health Organization (WHO) and the International Labour Organization (ILO).

If full-text versions of relevant studies are not available through those sources, we will contact the corresponding authors of the respective publications.

### Search strategy

Based on the selected eligibility criteria, search strategies were developed using Medical Subject Headings (MeSH) when applicable with combination of keywords based on multi-dimensional precarious employment definitions [28–30] and free text terms related to three main headings, namely, “migrant” (Population), “precarious employment” (Exposure), and “mental health” (Outcome). The draft search strategies for the respective electronic databases are provided in Additional file 2.

### Study records

#### Data management

EndNote X8.2 (Thomson Reuters) will be used for data management.

### Data Collection, selection process, and extraction

All articles identified from the literature search will be independently screened by two reviewers. First, titles and abstracts of articles returned from initial searches will be screened based on the eligibility criteria outlined above. Second, full texts will be examined in detail and screened for eligibility. Third, references of all considered articles will be hand-searched to identify any relevant report missed in the search strategy. Any disagreements will be reconciled by discussion to meet a consensus, if necessary. A flow chart showing details of studies included and excluded at each stage of the study selection process will be provided.

### Data collection

From each included study, using a standardized form, two reviewers will independently extract data on the following variables:

- First author
- Year of publication
- Location of the study
- Study design
- Study period
- Study population including number of participants
- Type of precarious employment (exposure)
- Outcome (s)
- Main results/effect estimates

A third reviewer will be consulted for resolution in case of disagreement. If relevant data cannot be extracted from the publications, we will contact the corresponding author of the respective manuscript.

Included studies will be grouped based on study design. The results of the quantitative studies will be summarized based on descriptive statistics in terms of frequencies, percentages, means, medians, relative risks (RRs), or odds ratios (ORs) with 95% confidence intervals where possible. The results of the qualitative studies will be summarized based on descriptive characteristics as well as main themes and subthemes.

### Risk of bias and quality assessment

For quantitative studies, two reviewers will independently rate the quality of the included studies with respect to methodological criteria such as selection of study participants, ascertainment of exposure and outcome, or case definition using the Newcastle-Ottawa Quality Assessment Scale (NOS) [31]. Discrepancies between the reviewers will be reconciled by consensus. Finally, each study will receive a score for low (0–3 points), moderate (4–6), or high quality (7–9).

To assess the quality of qualitative studies, the Critical Appraisal Skills Programme (CASP) for Evaluating Qualitative Research will be used [32]. It comprises ten questions, each of which is answered with “yes,” “no,” or “cannot tell”. A study will be branded a low-quality study if one or two of the first questions are marked as “no” or “cannot tell”. The CASP checklist has been used previously in systematic reviews of qualitative studies with a similar scope than the review presented here [33] (Additional file 3).

### Data analysis and synthesis

The results of the planned systematic review will be reported narratively using text and a table presenting the following variables for each included study: first author, year of publication, study design, study location, study period, study population and number of participants, type of precarious employment (exposure), outcome, and main results. If feasible and appropriate, data points from primary observational studies will be used to perform random effects meta-analyses. Since heterogeneity is expected a priori, we will estimate summary estimates (e.g., relative risks) its 95% confidence interval using the random effects model. The random effects model assumes the study prevalence estimates follow a normal distribution, considering both within-study and between-study variation. Forest plots will be used to visualize the extent of heterogeneity among studies. We will quantify statistical heterogeneity by estimating the variance between studies using the *I*^2^ statistics [34]..

A thematic analysis will be undertaken specifically for synthesis of qualitative studies [35]. It consists of three stages of analysis. A software program (MAXQDA, 2018 software for Windows) will be used to assist researchers in the thematic analysis process. An initial coding frame will be developed by one reviewer using the software program and will be checked by the second reviewer. In this process, the results of each study, including workers’ quotes, will be imported in full and verbatim into the software program. Findings, categories, and synthesized findings will be developed based on concepts. Each synthesized finding will be developed by at least two categorized findings, and each category will be developed by at least two findings [36]. As a result of this process, the coding frame will be completed, and themes and subthemes of the qualitative studies will be defined and described. Then, discussion and data examination will take place among the reviewers for combining the results of qualitative and quantitative studies. Finally, a two-stage meta-synthesis will be conducted. First, a form will be developed from qualitative studies based on the subthemes identified in these studies; this process will also occur for quantitative studies but will in a different way. Second, the results from this parallel synthesis in juxtaposition will be narratively written using text, figures, and tables. The relationships between outcomes both among and between retrieved studies will be discovered and elaborated.

### Additional analyses

To evaluate the potential of publication bias across studies, we will draw and evaluate funnel plots [37].

## Discussion

This systematic review will provide a thorough evaluation on how precarious employment affects migrant workers’ mental health. This systematic review of the existing qualitative and quantitative studies provides information on the following: the frequency of precarious employment among migrants; the association between precarious employment and mental health, including its direction and effect size; and processes underlying the relationship between precarious employment and migrant workers’ health, identified through the use of a scientifically valued review method. Thus, the review will provide evidence-based explanatory results about the association between precarious employment and migrant workers’ mental health.

However, we can foresee some sources of bias. At study level, the assessment of mental health outcomes as not trivial and is likely to vary between studies. While some studies may use clinical records for outcome assessment, other studies may use a questionnaire asking participants for self-reports of symptoms or if they ever had a doctor’s diagnosis of the outcome of interest. Similarly, given the already mentioned multi-dimensional character and various definitions of precarious employment [28–30], there may also be a certain degree of heterogeneity between studies with respect to the definition or operationalisation of precarious employment. In addition, the lack of gray literature might put our review at risk of bias. The gray literature, such as reports by companies in related fields, governmental and nongovernmental organizations, and conference abstracts, is defined as research that is not published in the form of articles in peer-viewed scientific journals, which provides a quality check during the submission process. In this process, it may also be possible to discover problems related to the results of studies [38]. Nevertheless, conference programs usually contain only a summary of each study, which does not allow for appropriate screening or evaluation [39]. Furthermore, all conference abstracts are not published in peer-viewed journals, which may cause “file-drawer” problems. The literature in languages other than English might be another foreseeable source of bias as there may be relevant publications in other languages, e.g., Chinese. However, previous systematic and meta-analysis studies indicate that the exclusion of studies published in languages other than English tend to have a small estimated effect size [40]. Moreover, we will also include literature published in three other languages (Spanish, German, or Turkish) as a criterion of inclusion, which decreases the risk of bias. Generally, studies with significant results are more likely to be published than are studies with non-significant results, which are defined as a publication bias. However, we address publication bias through funnel plots [41]. Lastly, limiting the review to studies published from 1970 onwards may prevent us from identifying relevant articles published before this date. The rationale behind this time limit is, however, that the term “precarious employment” has emerged mostly during the last decades and that we are confident that including the last 50 decades will be more than sufficient. Beyond that, there is even good reason to assume that what has been regarded as “precarious (employment/work)” more than 50 ago is very hard to compare to what is meant nowadays when talking about “precariousness.”

It is important to prevent mental health problems among workers. This review will be the first to date to provide scientifically valuable data in related fields. Thus, the results from the systematic review can be of great interest to labor and health professionals, stakeholders, policy makers, labour unions, and non-governmental organizations. Our findings may encourage and impel related policy makers to establish human focused, safe and healthy work environments, and workplace conditions. Our findings also might increase awareness among public citizens, including migrant workers, and professionals in the workplace (e.g., physicians, nurses, psychologists) about precarious work conditions and their effect on mental health, which may help to improve workers’ health and well-being. Additionally, the data may be used to develop a program to impede the side effects of precarious employment through the inclusion of occupational health experts in the workplace. Furthermore, the results may also guide researchers to the topics that need to be investigated in relevant fields in the future. The results of the review will be disseminated to the public through publications, conferences, and meetings.

## Supplementary information

**Additional file 1.** PRISMA-P 2015 Checklist

**Additional file 2.** Search Strings for the Databases

**Additional file 3.** The Critical Appraisal Skills Programme Checklist

## Data Availability

Not applicable

## Abbreviations

CASP: Critical Appraisal Skills Programme Checklist
ILO: International Labour Organization
MeSH: Medical Subject Headings
NOS: The Newcastle-Ottawa Quality Assessment Scale
PECO: Population, Exposure, Comparator, and Outcome Framework
PRISMA-P: Preferred Reporting Items for Systematic Review and Meta-Analysis-Protocol
PROSPERO: The International Prospective Register of Systematic Review
WHO: World Health Organization
