# Supplementary material for: Precarious employment and migrant workers’ mental health: a protocol for a systematic review of observational studies
Source: Syst Rev. 2020 Mar 7;9:50. doi: 10.1186/s13643-020-01313-w (PMC7060985; doi:10.1186/s13643-020-01313-w)
Supplement: Supplementary file 2 — Additional file 2. Search Strings for the Databases [file 13643_2020_1313_MOESM2_ESM.docx]

**Search Strings for the Databases**

**1. Pubmed/Medline**

| Search (insecur* OR layoff* OR nonstandard OR non-standard OR outsourc* OR “outsourced services”[MeSH Terms] OR “outsourced services” OR “part time” OR part-time OR downsizing OR “personnel downsizing”[MeSH Terms] OR “personnel downsizing” OR precarious OR “short-term” OR “staffing agency” OR temporar* OR unprotected OR “work condition” OR “work conditions” OR “workplace rights” OR informal OR “inconvenient hour” OR “inadequate wage” OR “insufficient wage” OR “poor wage” OR “workplace right” OR atypical OR casual OR contingent OR discontinuity OR disempowerment OR “flexible hour” OR “flexible hours” OR “inconvenient hours” OR “inadequate wages” OR “insufficient wages” OR “minimum wage” OR “minimum wages” OR “low wage” OR “low wages” OR “poor wage” OR “poor wages” OR “inadequate salary” OR “insufficient salary” OR “low salary” OR “poor salary” OR “minimum salary”) AND (employment[MeSH Terms] OR employment OR work[MeSH Terms] OR work OR Occupations[MeSH Terms] OR occupation* OR workplace[MeSH Terms] OR workplace OR Job*) AND (“emigrants and immigrants”[MeSH Terms] OR “emigrants and immigrants” OR “undocumented immigrants”[MeSH Terms] OR “undocumented immigrants” OR “transients and migrants”[MeSH Terms] OR “transients and migrants” OR foreigner* OR “foreign worker” OR “foreign workers” OR “foreign-born workers” OR immigrant* OR migrant* OR “immigrant worker” OR “immigrant workers” OR “migrant worker” OR “migrant workers” OR emigrant* OR “foreign-born worker” OR “oversea worker” OR “oversea workers”) AND (“mental health”[MeSH Terms] OR “mental health” OR “mental disorders”[MeSH Terms] OR “mental disorders” OR depression[MeSH Terms] OR depression OR “depressive disorder”[MeSH Terms] OR “depressive disorder” OR anxiety[MeSH Terms] OR anxiety OR “anxiety disorders”[MeSH Terms] OR “anxiety disorders” OR dyssomnias[MeSH Terms] OR dyssomnia* OR sleep[MeSH Terms] OR sleep OR “sleep initiation and maintenance disorders”[MeSH Terms] OR insomnia OR “burnout, psychological”[MeSH Terms] OR “burnout, psychological” OR “burnout, professional”[MeSH Terms] OR “burnout, professional” OR burn-out OR burnout OR “occupational stress” [MeSH Terms] OR “occupational stress” OR “job stress” OR “stress” OR “work-related stress”) Filters: Journal Article; Publication date from 1970/01/01 to 2019/08/31; English; German; Spanish; Turkish; MEDLINE |
| --- |

**2. Web of Science**

| \|  \| [**...More**](javascript:void(0))(ALL=("inconvenient hour" OR "inadequate wage" OR "insufficient wage" OR "poor wage" OR "workplace right" OR atypical OR casual OR contingent OR discontinuity OR disempowerment OR "flexible hour" OR "flexible hours" OR "inconvenient hours" OR "inadequate wages" OR "insufficient wages" OR "minimum wage" OR "minimum wages" OR "low wage" OR "low wages" OR "poor wage" OR "poor wages" OR "inadequate salary" OR "insufficient salary" OR "low salary" OR "poor salary" OR "minimum salary" OR insecur* OR layoff* OR nonstandard OR non-standard OR outsourc* OR "outsourced services" OR "part time" OR part-time OR downsizing OR "personnel downsizing" OR precarious OR "short-term" OR "staffing agency" OR temporar* OR unprotected OR "work condition" OR "work conditions" OR "workplace rights" OR informal) AND ALL=( employment OR work OR occupation* OR workplace OR Job*) AND ALL=("emigrants and immigrants" OR "undocumented immigrants" OR "transients and migrants" OR foreigner* OR "foreign worker" OR "foreign workers" OR "foreign-born workers" OR immigrant* OR migrant* OR "immigrant worker" OR "immigrant workers" OR "migrant worker" OR "migrant workers"OR emigrant* OR "foreign-born worker" OR "oversea worker" OR "oversea workers") AND ALL=( "mental Health" OR "mental disorders" OR depression OR "depressive disorder" OR anxiety OR "anxiety disorders" OR dyssomnia* OR sleep OR "sleep initiation and maintenance disorders" OR insomnia OR "burnout, professional" OR burn-out OR burnout OR "occupational stress" OR "job stress" OR "work-related stress" OR stress)) *AND* **DOCUMENT TYPES:** (Article)  **Refined by:** **LANGUAGES:** ( ENGLISH OR SPANISH OR GERMAN ) AND **DOCUMENT TYPES:** ( ARTICLE )  **Timespan:** 1970-2019. **Indexes:** SCI-EXPANDED, SSCI, A&HCI, CPCI-S, CPCI-SSH, ESCI \|  \| \| --- \| --- \| --- \| |
| --- | --- | --- | --- |

**3. PsycINFO**

| S5 = S1 AND S2 AND S3 AND S4  S1= MA "emigrants and immigrants" OR TX "emigrants and immigrants" OR MA "undocumented immigrants" OR TX "undocumented immigrants" OR MA "transients and migrants" OR TX "transients and migrants" OR TX "foreigner*" OR TX "foreign worker" OR TX "foreign-born worker" OR TX "immigrant*" OR TX "migrant*" OR TX "emigrant*" TX "foreign-born workers" OR TX "foreign workers" OR TX "oversea worker" OR TX "oversea workers" OR TX "immigrant worker" OR TX "immigrant workers" OR TX "migrant worker" OR TX "migrant workers"  AND  S2= MA employment OR TX "employment" OR MA work OR TX work OR MA occupations OR TX "occupation*" OR MA workplace OR TX "workplace"  AND  S3= TX "atypical" OR TX "casual" OR TX "contingent" OR TX "discontinuity" OR TX "disempowerment" OR TX "flexible hour" OR TX "flexible hours" OR TX "inconvenient hour" OR TX "inconvenient hours" OR TX "inadequate wage" OR TX "inadequate wages" OR TX "inadequate wages" OR TX "personnel downsizing" OR TX "precarious" OR TX "short-term" OR TX "staffing agency" OR TX "temporar*" OR TX "unprotected" OR TX "work condition" OR TX "work conditions" OR TX "workplace right" OR TX "workplace rights" OR TX "informal" OR TX "minimum salary" OR TX "insecur*" OR TX "layoff*" OR TX "nonstandard" OR TX "non-standard" OR TX "outsourc*" OR MA "outsourced services" AND TX "outsourced services" OR TX "part time" OR TX "part-time" OR TX "downsizing" OR MA "personnel downsizing" OR TX "insufficient wage" OR TX "insufficient wages" AND TX "minimum wage" OR TX "minimum wages" OR TX "low wage" OR TX "low wages" OR TX "poor wage" OR TX "poor wages" OR TX "inadequate salary" OR TX "insufficient salary" OR TX "low salary" OR TX "poor salary" OR TX "minimum salary" OR TX "insecur*" OR TX "layoff*" OR TX "nonstandard" OR TX "non-standard" OR TX "outsourc*" OR MA "outsourced services" AND TX "outsourced services" OR TX "part time" OR TX "part-time" OR TX "downsizing" OR MA "personnel downsizing" OR TX "personnel downsizing" OR TX "precarious" OR TX "short-term" OR TX "staffing agency" OR TX "temporar*" OR TX "unprotected" OR TX "work condition" OR TX "work conditions" OR TX "workplace right" OR TX "workplace rights" OR TX "informal"  AND  S4= MA "mental health" OR TX "mental health" OR MA "mental disorders" OR TX "mental disorders" OR MA "depression" OR TX "depression" OR MA "depressive disorder" OR MA "depressive disorder" OR MA "anxiety" OR TX "anxiety" OR MA "anxiety disorders" OR TX "anxiety disorders" OR MA "dyssomnias” OR TX "dyssomnias” OR MA “sleep” OR TX “sleep” OR MA “sleep initiation and maintenance disorders” OR TX “insomnia” OR MA “burnout, psychological” OR TX “burnout, psychological” OR MA “burnout, professional” OR TX “burnout, professional” OR TX “burn-out” OR TX “burnout” OR MA “occupational stress” OR TX “occupational stress” OR TX “job stress” OR TX “work-related stress” OR TX “stress”  Limiters - Publication Year: 1970-2019; Published Date: 19700101-20190831; Publication Type: Peer Reviewed Journal; Document Type: Journal Article |
| --- |
