# Supplementary material for: Precarious employment and migrant workers’ mental health: a protocol for a systematic review of observational studies
Source: Syst Rev. 2020 Mar 7;9:50. doi: 10.1186/s13643-020-01313-w (PMC7060985; doi:10.1186/s13643-020-01313-w)
Supplement: Supplementary file 3 — Additional file 3. The Critical Appraisal Skills Programme Checklist [file 13643_2020_1313_MOESM3_ESM.docx]

Additional file 3: The Critical Appraisal Skills Programme Checklist

|  | | Yes | Can’t tell | | No |
| --- | --- | --- | --- | --- | --- |
| Section A  Are the Results valid? | 1. Was there a clear statement of the aim of the research?   *Comments:* |  | |  |  |
|  | 1. Is a qualitative methodology appropriate?   *Comments:* |  | |  |  |
|  | 1. Was the research design appropriate to address the aims of the research?   *Comments:* |  | |  |  |
|  | 1. Was the recruitment strategy appropriate to the aims of the research?   *Comments:* |  | |  |  |
|  | 1. Was the collected in a way that addresses the research issue?   *Comments:* |  | |  |  |
|  | 1. Has the relationship between researcher and participants been adequately considered?   *Comments:* |  | |  |  |
| Section B  What are the Results? | 1. Have ethical issues been taken into consideration?   *Comments:* |  | |  |  |
|  | 1. Was the data analysis sufficiently rigorous?   *Comments:* |  | |  |  |
|  | 1. Is there a clear statement of findings?   *Comments:* |  | |  |  |
| Section C  Will the results help locally? | 1. How valuable is the research?   *Comments:* |  | |  |  |
